# Genetic Diversity of Canine Circovirus Detected in Wild Carnivores in Serbia

**DOI:** 10.3390/vetsci12060515

**Published:** 2025-05-24

**Authors:** Damir Benković, Jakov Nišavić, Nenad Milić, Dejan Krnjaić, Isidora Prošić, Vladimir Gajdov, Nataša Stević, Ratko Sukara, Martina Balać, Andrea Radalj

**Affiliations:** 1 Veterinary Specialist Institute “Sombor”, 25000 Sombor, Serbia; 2 Department of Microbiology, Faculty of Veterinary Medicine, University of Belgrade, 11000 Belgrade, Serbia; jakovmoni@vet.bg.ac.rs (J.N.); nenadmilic@vet.bg.ac.rs (N.M.); dejan.krnjaic@vet.bg.ac.rs (D.K.); isidora.prosic@vet.bg.ac.rs (I.P.); 3 Scientific Veterinary Institute “Novi Sad”, 21113 Novi Sad, Serbia; vladimir.g@niv.ns.ac.rs; 4 Department of Infectious Diseases and Diseases of Bees, Faculty of Veterinary Medicine, University of Belgrade, 11000 Belgrade, Serbia; natasas@vet.bg.ac.rs; 5 Institute for Medical Research, University of Belgrade, 11000 Belgrade, Serbia; ratko.sukara@imi.bg.ac.rs; 6 Faculty of Mechanical Engineering, University of Belgrade, 11000 Belgrade, Serbia; mbalac@mas.bg.ac.rs

**Keywords:** CanineCV, golden jackal, molecular characterization, recombination, red fox, wildlife

## Abstract

Canine circovirus (CanineCV) is an emerging virus of interest in both domestic and wild carnivores that is scarcely reported in southeastern Europe. This study examined the presence, genetic diversity, and evolutionary characteristics of CanineCV in red foxes (*Vulpes vulpes*) and golden jackals (*Canis aureus*) from northwestern Serbia, a region marked by expanding mesopredator populations overlapping with human habitats. Out of 98 sampled animals, circoviral DNA was detected in 31.6%. Jackals were mostly positive for CanineCV genotype 4, while genotype 5, associated with wild carnivores, was dominant in foxes. Mixed genotype 4/genotype 5 infections were only found in jackals. Phylogenetic and haplotype analyses indicated that most jackal-derived CanineCV strains clustered along sequences from Europe, Africa, and the Americas, while genotype 5 sequences grouped separately from other genotype representatives. A recombinant strain was identified as a divergent lineage, and several sequences showed evidence of recombination between *Rep* and *Cap* genes. Despite *Cap* protein amino acid differences, purifying selection dominated, suggesting functional constraints on viral evolution. The results indicate that jackals may act as recombination hotspots and bridging hosts between viral lineages. This study provides insight into the molecular epidemiology of CanineCV in the Balkans, highlighting the importance of ongoing surveillance.

## Simple Summary

Wild carnivore species such as golden jackals and red foxes are increasingly present near human settlements which is concerning since these animals represent reservoirs of diverse pathogens. Canine circovirus is an emerging virus known to cause various symptoms in domestic and wild carnivores and to complicate other infections. This study examined the presence and genetic characteristics of canine circovirus strains in these animals in an area in Northwest Serbia presented by diverse landscapes, transboundary locations, and overlapping between domestic and wild carnivore populations. The virus was detected in 31.6% of tissue samples, with jackals more commonly carrying strains typically found in domestic dogs, and foxes harboring a particular variant detected in wildlife. In several jackal samples, both virus variants were simultaneously detected, implying these animals may serve as hosts for viral mixing. Genetic examinations proved the presence of diverse virus strains, and genetic recombinations, revealing viral evolution. These results highlight the role of wild carnivores, especially jackals, as potential bridges between wildlife and domestic animals in viral transmission and evolution. Ongoing monitoring is essential for an improved understanding of the spreading of canine circovirus, evolutionary patterns, and potential risks to animal health, particularly in areas where domestic and wildlife populations increasingly overlap.

## 1. Introduction

The recent expansion of the golden jackal (*Canis aureus*) population across southeastern and central Europe has raised interest in their potential role as disease carriers, particularly given their tendency to roam peri-urban areas in search of food [1,2]. Similarly, the red foxes (*Vulpes vulpes*), as the most widely distributed wild carnivore globally, are highly adaptable and often found near human settlements, which is especially important due to their known role in disease transmission [3,4].

Viruses from the *Circoviridae* family are non-enveloped and share a unique genome consisting of a single-stranded circular DNA of ~2 kb with two segments: the conserved *Rep* segment, essential for viral replication and variable *Cap* segment which encodes the viral capsid [5,6]. Canine circovirus (CanineCV) was first discovered in 2012 in asymptomatic dogs but has since been connected to cases of vasculitis, hemorrhagic diarrhea, respiratory disease, and lymphadenitis, as well as neurological manifestations in foxes [7,8,9,10]. Similarly to porcine circoviruses, CanineCV is a relatively common finding in clinically healthy individuals; however, it may cause disease or further exacerbate other infections, especially those caused by the canine parvovirus [11]. Circoviruses are generally stable in the environment, which facilitates the possibility of indirect transmission within susceptible populations [10,12].

The role of CanineCV in disease pathogenesis remains unclear, particularly given its transmissibility among dogs and wild carnivores, which is especially concerning in areas where their habitats overlap [13,14]. Molecular characterization studies of strains of CanineCV are mostly based on whole viral genome analysis, and there are numerous propositions on their classification. Recent studies show that CanineCV is divided into six genotypes, with genotype 1 being most common in dogs and wolves, genotypes 2–4 are mostly from Asia, genotype 6 consists of Iranian strains, while genotype 5 comprises strains found in foxes, and is also called FoxCV, which was until recently considered to be a separate species [15,16,17,18,19,20]. Additionally, as indicated by multiple studies, genetic recombination among CanineCV strains appears to be common, highlighting the need for continuing surveillance of viral evolution [9,14,21,22]. CanineCV has emerged as a pathogen of interest not only in domestic dogs but also in wild carnivores; however, available data concerning this subject are scarce for the Balkans. This region, particularly Serbia, is a transition zone between central and southeastern Europe and acts as a natural corridor for wildlife movement. Having this in mind, our study aimed to characterize CanineCV detected in these expanding mesopredator populations and provide new insight into the circulation and molecular diversity of CanineCV in this understudied area.

## 2. Materials and Methods

### 2.1. Sampling

The samples were collected from hunted animals during regular monitoring of the efficacy of oral rabies vaccination of wild carnivores in Serbia during 2022. Samples included blood, lungs, liver, spleen, kidney, intestine, and medulla from 98 animals (45 jackals and 53 foxes), and these were immediately processed upon arrival in the laboratory. Sampling was performed during pathoanatomical examination of the animals, and none of the animals displayed pathomorphological lesions suggestive of infectious disease. Automated nucleic acid extraction from 80 µL of homogenized tissue was performed using IDEAL32 extraction robot and ID Gene MagFast Extraction kit (Innovative Diagnostics, Grabels, France). The extracts were stored at −80 °C pending further analysis.

Samples were obtained from an area in Northwest Serbia with diverse landscapes, transboundary location, and increasing overlap between domestic and wild carnivore populations. The study area covered 2.420 km^2^, namely from the following 17 locations: Apatin (45.67197° N, 18.97986° E), Bački Monoštor (45.792141° N, 18.93309° E), Kolut (45.892139° N, 18.925131° E), Panonija (45.746441° N, 19.523661° E), Lalinske Livade (45.5058662° N, 19.2597368° E), Telečka (45.804766° N, 19.38826° E), Stara Moravica (45.8693425° N, 19.4666165° E), Čonoplja (45.812126° N, 19.248848° E), Bačka Topola (45.8355451° N, 19.5947065° E), Kozara (45.83015° N, 18.93715° E), Sonta (45.555332° N, 19.066082° E), Odžaci (45.4927° N, 19.24492° E), Kula (45.622452° N, 19.537297° E), Pačir (45.896571° N, 19.400683° E), Sivac (45.711108° N, 19.387221° E), Kruščić (45.625136° N, 19.360849° E), and Vrbas (45.583568° N, 19.68652° E).

### 2.2. Real-Time PCR

The samples underwent examination using two in-house Real-time PCR protocols, designed as duplex PCRs with an endogenous control: one targeting a conserved region of the CanineCV genome (generic detection), and another using genotype 5-specific primers and probe, as previously described [7,8]. In this setup, samples testing positive with the genotype 5 assay were provisionally considered genotype 5, while those positive only in the general assay were classified as CanineCV-positive. The final genotype assignment was determined through sequencing and phylogenetic analysis. For all samples, we included an endogenous extraction and PCR inhibitor control, following a protocol targeting the β-actin housekeeping gene [23]. In our experiments, reactions were conducted in 20 µL volumes using the Luna Universal Probe qPCR Master Mix (New England Biolabs, Ipswich, MA, USA). The components were added in the following volumes: 2 µL of nuclease-free water, 10 µL of master mix. For CanineCV and genotype 5, we used 0.8 µL each of forward and reverse primers, along with 0.4 µL of the probe. For the β-actin housekeeping gene, we employed 0.4 µL of each forward and reverse primers and 0.2 µL of the probe. Additionally, 5 µL of nucleic acid extract was included. The thermal profile for all reactions consisted of the following steps: an initial denaturation at 95 °C for 1 min, followed by 45 cycles of 95 °C for 15 s and 60 °C for 30 s. Fluorescence was detected at the annealing/elongation temperatures using the FAM channel for CanineCV, and the HEX channel for β-actin. All tests were conducted on an AriaMx thermal cycler (Agilent Technologies, Santa Clara, CA, USA). Amplification curve analysis was performed using the “auto-baseline” setting.

### 2.3. Sequencing

Whole genome sequencing of the detected circovirus strains was conducted using the Sanger sequencing method, with primers designed to amplify overlapping segments as previously described by Piewbang et al. [9]. Positive samples were sent to Macrogen Europe Laboratory in the Netherlands for sequencing. The products were sequenced in both directions, and consensus sequences were generated using MEGA XI software version 11.0.13. All sequences were analyzed using BLASTn (http://blast.ncbi.nlm.nih.gov, accessed on 10 March 2024). Following the initial analysis of the obtained data, nine high-quality sequences, encompassing nearly the entire genome (partial *Rep* gene and complete *Cap* gene, 1902 bp in total), were chosen as representatives for further bioinformatic analysis.

### 2.4. Phylogenetic Analysis

Whole genome sequences of CanineCV were retrieved from GenBank and aligned with sequences acquired in this study using the MUSCLE algorithm incorporated in MEGA XI [24]. Phylogenetic analysis including all obtained sequences and CanineCV representatives from GenBank was performed using the Neighbor-joining method. Subsequently, the appropriate substitution model for genotyping analysis was selected using “Find Best DNA/Protein Models” option. The evolutionary history was inferred using the Maximum Likelihood method based on the GTR + G + I substitution model. The first trial included 107 full genome sequences of CanineCV from domestic dogs and wild carnivores. The phylogenetic relationships among CanineCV genotype 5 representatives were evaluated by comparing them with 49 analogous sequences from GenBank. The reliability of the inferred clades for each mentioned dataset was evaluated by performing 1000 bootstrap replicates.

### 2.5. Nucleotide and Amino Acid Sequence Analysis

Nucleotide similarities between the obtained and representative sequences retrieved from GenBank were calculated using BioEdit software v. 7.2. Nucleotide substitution rates for the *Cap* gene were analyzed in MEGA XI. The complete *Cap* gene sequences from this study were aligned with reference sequences for both CanineCV (NC_020904) and CanineCV genotype 5 (KP260927), translated into amino acids and analyzed using BioEdit.

### 2.6. Recombination Analysis

The investigation for probable recombination breakpoints employed GARD [25], and characterization of the potential recombinants was conducted with RDP4 with the following methods enabled: RDP, GENECONV, BootScan, MaxChi, Chimaera, SiScan, and 3Seq [26]. Method configurations were tailored following the guidelines in the RDP4 manual, and only recombination events identified by three different methods with a *p*-value below 0.001 (*p* < 0.001) were considered credible. Recombination was assessed using RDP4 [26]. 

### 2.7. Haplotype Diversity

Haplotype diversity (Hd), nucleotide diversity (π), and average number of nucleotide differences (k) were analyzed using DnaSP software version 6.0 [27]. PopART software version 1.7 was used to generate a Templeton, Crandall, and Sing (TCS) haplotype network [28].

### 2.8. Selection Pressure Analysis

An analysis of selection pressure on the *Cap* protein of nine CanineCV strains from our study was evaluated by different methods using Datamonkey (http://www.datamonkey.org, accessed on 12 March 2025), based on the ratios between non-synonymous and synonymous substitution rates (dN/dS) [29,30]. The methods included Single-likelihood ancestor counting (SLAC), mixed effects model of evolution (MEME), fixed-effects likelihood (FEL), and fast unconstrained Bayesian approximation (FUBAR). Sites were regarded as positive (diversifying) selection when the *p*-value was below 0.1 in SLAC, MEME, and FEL, and the posterior probability was above 0.9 in FUBAR. The sites under diversifying selection were validated when at least two methods detected the mentioned values.

## 3. Results

### 3.1. Real-Time PCR

PCR results revealed 31/98 (31.6%) samples positive for the presence of circovirus DNA. Among these, 51.6% were single infections with CanineCV genotype 5 (confirmed by genotype-specific PCR), 35.5% were positive for CanineCV by general detection (without genotype assignment), and 12.9% were mixed infections with both genotype 5-specific and general CanineCV signals.

**Figure 1:**
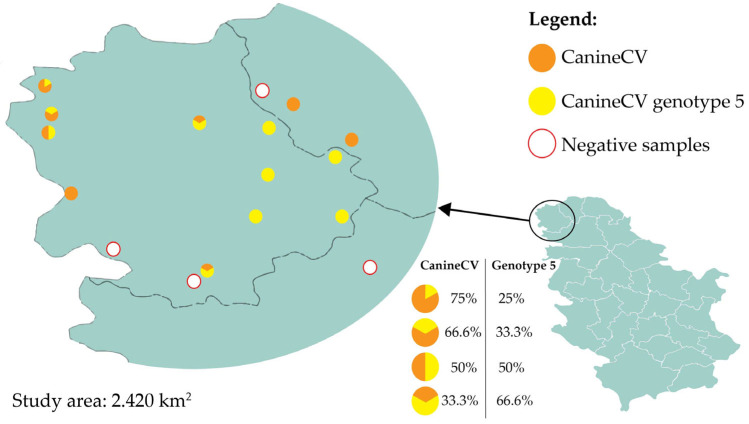
Sampling locations and proportion of CanineCV and CanineCV genotype 5 positive samples in wild carnivores. Percentages are rounded to one decimal place; totals may not sum to exactly 100% due to rounding.

Jackal samples were dominantly positive in the general CanineCV assay, while red fox samples mostly tested positive for genotype 5. Mixed infections were detected solely in jackal samples (Table 1). Across 17 locations, circovirus positivity ranged from 0% (Sonta, Odžaci, Pačir, and Vrbas) to 100% (Apatin, Panonija, Stara Moravica, Bačka Topola, Telečka, Kula, Sivac, and Kruščić). Samples from other locations showed different levels of positivity (Figure 1).

### 3.2. Phylogenetic Analysis

**Figure 2:**
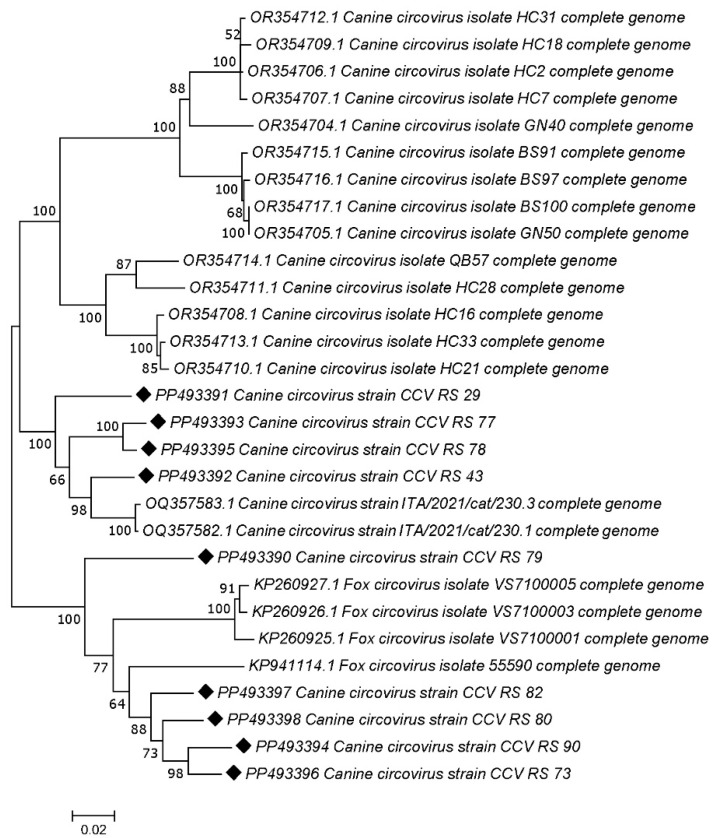
Neighbor-joining phylogenetic tree based on the nucleotide sequences of 9 wild carnivore CanineCV strains obtained in this study (marked) and 17 representative sequences retrieved from GenBank. The numbers associated with the nodes represent the percentage of 1000 bootstrap iterations supporting the nodes (only percentages > 50% are shown). GenBank accession numbers, strain names are indicated.

The phylogenetic analysis revealed that five samples isolated from jackals (accession numbers: PP493390, PP493398, PP493397, and PP493394) cluster with CanineCV genotype 5 (designated as FoxCV in earlier studies) along with the fox-derived sample from this study PP493396. The other four jackal samples (accession numbers: PP493391, PP493393, PP493395, and PP493392) clustered with CanineCV strains as shown in Figure 2.

**Figure 3:**
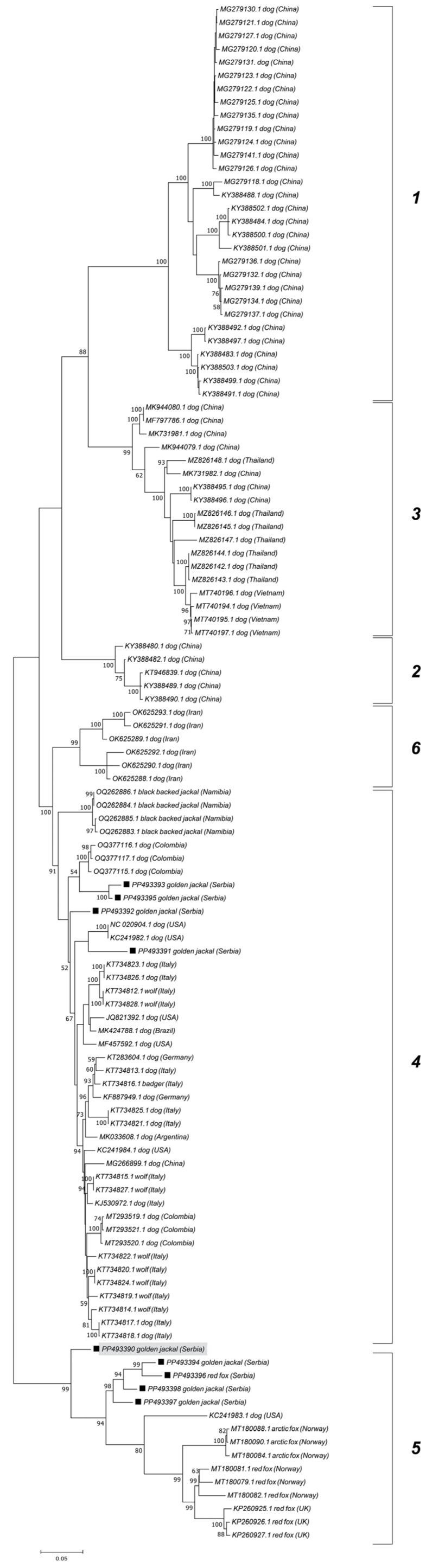
Maximum Likelihood phylogenetic tree based on the nucleotide sequences of 9 wild carnivore CanineCV strains obtained in this study (marked) and 107 sequences retrieved from GenBank representing each genotype. The numbers associated with the nodes represent the percentage of 1000 bootstrap iterations supporting the nodes (only percentages > 50% are shown). GenBank accession numbers, genotypes, hosts, and the country of origin are indicated. Recombinant jackal-derived sequence is marked gray.

**Figure 4:**
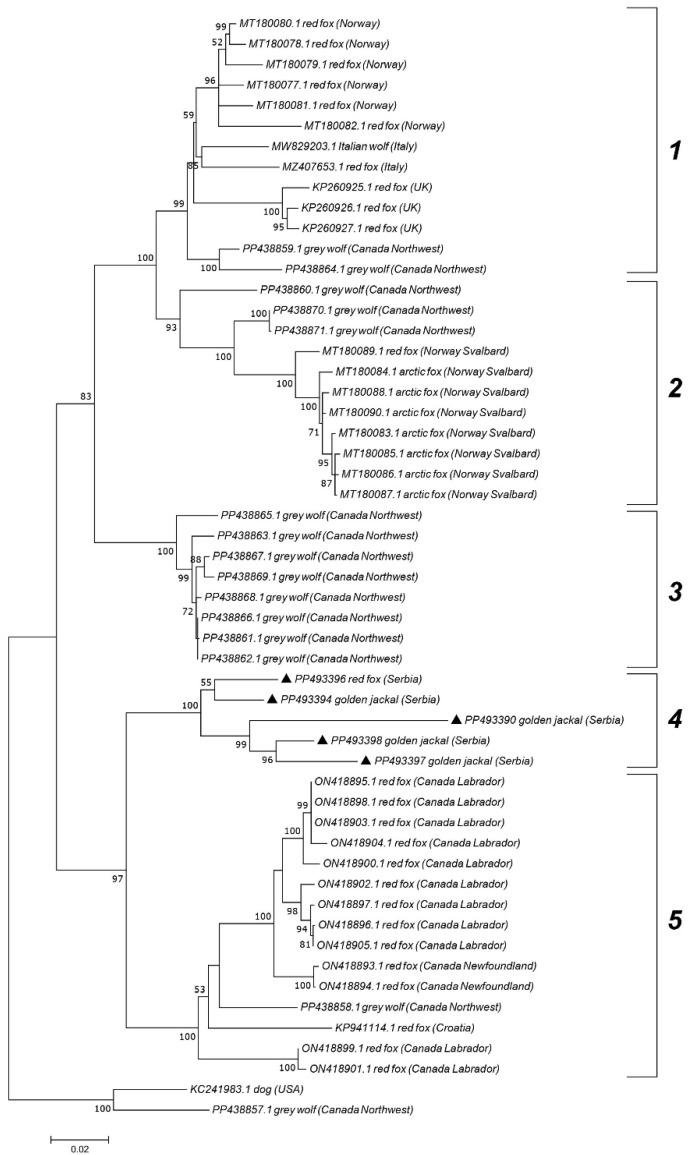
Maximum Likelihood phylogenetic tree based on the nucleotide sequences of 5 wild carnivore CanineCV strains (genotype 5) obtained in this study (marked) and 49 sequences retrieved from GenBank representing different clades. The numbers associated with the nodes represent the percentage of 1000 bootstrap iterations supporting the nodes (only percentages > 50% are shown). GenBank accession numbers, clades, hosts, and the country of origin are indicated.

The ML phylogenetic trees show the segregation of the examined CanineCV sequences into established genotypes (Figure 3 and Figure 4). Jackal-derived sequences from this study (PP493391, PP493393, PP493395, and PP493392) grouped into a clade corresponding to genotype 4, as reported by Urbani et al. [17]. Genotype 4 includes strains from Europe, the USA, South America, and Africa, while genotypes 1, 2, and 3 comprise Asian CanineCV sequences. Genotype 6 comprises Iranian sequences as recently demonstrated by Beikpour et al. [18]. The remaining five jackal and fox-derived sequences from this study, clustered within genotype 5, a lineage primarily associated with wildlife. All sequences from Serbia assigned to genotype 5 formed a distinct cluster, with the exception of PP493390, which diverged from the group.

Genotype 5 sequences, primarily associated with wild carnivores, were further subdivided into five clades, displaying geographic separation. Serbian genotype 5 sequences from jackals (PP493390, PP493394, PP493396, and PP493398) and one red fox (PP493397) grouped into clade 4. Clade 1 primarily consisted of strains from Norway, Italy, and the UK, while clade 2 was represented by strains from Svalbard. Clade 5 included sequences from Canada (Labrador and Newfoundland) and one sequence from Croatia. Sequences from the Canadian Northwest Territories created clade 3 but were also found in all clades except clade 4.

### 3.3. Genetic and Amino Acid Analysis

Nucleotide similarities among all sequences from this study ranged from 83.8% to 98.3%. The genotype 4 sequences (from jackals) exhibited 92.1–98.3% similarity among themselves, while genotype 5 sequences (from both jackals and foxes) showed 91.6–96.3% similarity. When compared to sequences from GenBank, the similarities of Serbian genotype 4 sequences ranged from 84.3% to 96.7%. Overall, the highest similarities were detected with analogous sequences from Italy, Germany, Africa, the USA, and South America, with notable differences from strains from China, Taiwan, and Vietnam. Furthermore, Serbian genotype 5 sequences exhibited similarities with other GenBank sequences ranging from 87.1% to 93.2%, being most similar to strains from red foxes in Norway and most dissimilar to those from red foxes in the UK and arctic foxes in Svalbard.

The nucleotide substitution matrix for the *Cap* gene, estimated under the General Time Reversible model (+G+I) with gamma-distributed rate variation and 25% invariable sites, reveals a higher rate of transitions over transversions (Table 2), nucleotide frequencies of A (31.60%), T (21.33%), C (25.32%), and G (21.74%), and a maximum log likelihood of −2712.384.

The 270-amino-acid capsid protein of the analyzed strains was encoded by the *Cap* gene located at nucleotide positions 972 to 1784. Capsid protein sequences of genotype 4 strains (from jackals) showed 93.7–97.7% similarity among themselves and 94.8–97.4% similarity to the reference sequence (NC_020904). In contrast, genotype 5 strains (from both jackals and foxes) showed 87.4–95.1% similarity among themselves and 86.6–92.5% similarity to the corresponding reference sequence (KP260927). The number of amino acid position variations in the *Cap* protein sequences of genotype 4 and genotype 5, compared to analogous reference sequences, was 24 and 47, respectively. Genotype 4 sequences shared a conserved mutation at position 195, and all strains (PP493391, PP493392, PP493393, and PP493395) exhibited a substitution from Alanine (A) to Threonine (T) relative to the reference strain. In genotype 5 sequences, seven conserved amino acid changes were identified at positions 14, 28, 102, 103, 113, 148, and 257, all uniformly mutated across all five strains in our study compared to the reference sequence (Supplementary Tables S1 and S2a,b). 

### 3.4. Recombination Analysis

The GARD analysis revealed the presence of a potential recombination breakpoint between *Rep* and *Cap* genes around position 900. Recombination analysis using RDP4 showed the potential recombinant origin of two genotype 4 and four genotype 5 strains, originating from multiple recombination events and in two separate genome positions. Only recombination events supported by at least three different methods (RDP, GENECONV and BootScan,) with *p*-values below 0.001 were considered valid. One fox and five jackal-derived sequences (PP493396, PP493398, PP493397, PP493394, PP493393, and PP493395) likely originated from recombination between strains detected in the present study and occurred approximately between positions 900 and 1400. For the jackal-derived sample (PP493390), a potential insertion was noted between positions 500 and 1000 and included a close relative to the other genotype 5 sequences and another foreign strain as parental strains.

### 3.5. Haplotype Diversity

After analyzing alignments containing both the partial *Rep* and complete *Cap* sequences from this study, a total of 145 haplotypes were detected, with an Hd value of 1.000 ± 0.001. The nucleotide diversity (π) was 0.131 ± 0.001, and the average number of nucleotide differences (k) was 249.324. Sequences from Serbia were divided into nine haplotypes, with Hd, π, and k values of 1.000 ± 0.052, 0.101 ± 0.009, and 192.667.

**Figure 5:**
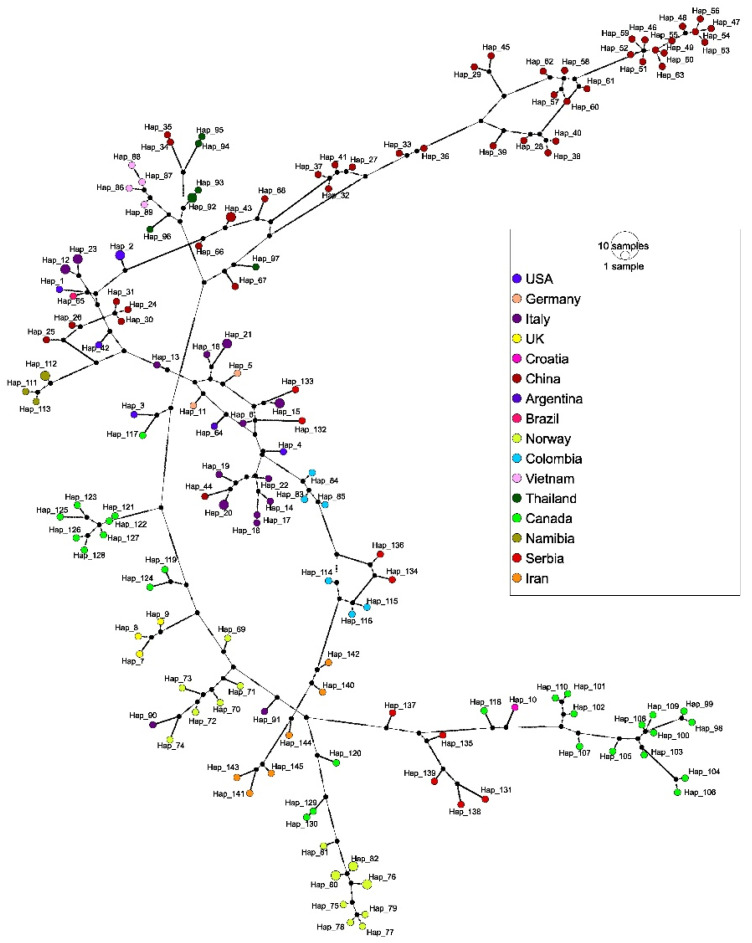
The haplotype network based on 158 CanineCV sequences (1902 bp) previously used for phylogenetic analysis. The geographical distribution of haplotypes is indicated by the color scheme in the legend. The circle size is proportional to haplotype frequency and the number of mutations between haplotypes is shown by hatch marks.

The TCS network revealed 145 haplotypes and supported the results of the phylogenetic analysis (Figure 5). Each sequence from this study represented a unique haplotype (Hap_131–Hap_139) with up to 199 mutational differences. Jackal genotype 4 sequences Hap_136 (PP493395) and Hap_134 (PP493393) were separated by 31 mutations and grouped with haplotype representatives from dogs in Colombia. Similarly, jackal sequences Hap_132 (PP493391) and Hap_133 (PP493392) clustered with Italian sequences from dogs (Hap_6) and wolves (Hap_15), separated by 100 and 63 mutations, respectively. Genotype 5 sequences from jackals (Hap_131, Hap_135, Hap_138, and Hap_139) grouped with other genotype 5 representatives from Croatia and Canada. Consistent with the phylogenetic tree, Hap_131 (PP493390) stemmed as the most divergent haplotype and was linked to this node by 100 mutations. In contrast, the other haplotypes within this cluster revealed less mutational differences, indicating that Hap_131 represents a divergent lineage. Furthermore, the red fox sequence Hap_137 (PP493396) lies on a separate branch within the network, with 29 mutations to the nearest node.

### 3.6. Selection Pressure

**Figure 6:**
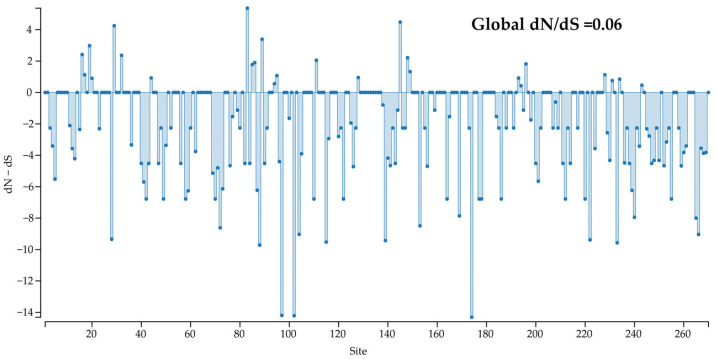
Selection pressure on the *Cap* protein of CanineCV strains: the global dN/dS ratio in the *Cap* coding sequence.

Selection pressure on the *Cap* protein of CanineCV strains was estimated, and negatively selected sites (dN − dS < 0) were predominantly observed across the coding region (Figure 6). Additionally, the global dN/dS ratio estimated in the *Cap* coding sequence using SLAC was lower than 1 (0.06), reflecting that most non-synonymous mutations are deleterious and are being removed by selection. The analysis revealed 35, 72, and 75 sites with statistically significant purifying (negative) selection according to SLAC, FEL, and FUBAR, respectively. Namely, up to 27.8% of *Cap* gene codons were predicted to be evolving under a purifying selection. Contrarily, significant diversifying (positive) selection was observed at one site (codon 83) by MEME, FEL, and FUBAR (Table 3).

## 4. Discussion

The subject of CanineCV detection and characterization has been increasingly revisited in the past several years since its diversity and global distribution in domestic dogs and wild canids warrant systematic investigations [10,16,19,31]. Retrospective analyses have shown the presence of CanineCV in samples dating back to 1996, while some bioinformatic analyses imply that foxes were the source of this virus [16,17]. Aside from the findings of Bexton et al. [8], which characterized CanineCV genotype 5 (marked as FoxCV) in animals with meningoencephalitis, most investigations report no correlation between virus positivity in wild animals and visible pathomorphological signs [16,32]. This aligns with our findings, which suggest subclinical infections in the sampled individuals, consistent with the typical profile of circovirus infections [12]. It is well-known that these infections often require additional factors to induce severe disease, as observed with porcine circoviruses (PCVs). However, confirming such cases can be particularly challenging when dealing with samples originating from wild animals [33].

CanineCV has been detected in several wildlife species in investigations published so far, including wolves, red and Arctic foxes, badgers, and black-backed jackals [13,14,16,17,19]. Our study focused on the most abundant populations of wild canids in Serbia and displayed a relatively high proportion of positive samples. The overall infection frequency in wildlife differs between studies, but these results cannot be directly compared due to differences in study areas and sample sizes. Available reports from Italy range from no evidence of fox infection to the detection in up to 5% of samples [13,16,34]. Regarding samples obtained from foxes, results similar to our findings were reported in Norway, the UK, and Canada, where genotype 5 was identified in 16.9% to 51.6% of sampled animals [8,17,32]. Also, certain authors report high infection frequencies in wolves, with detection rates of up to 45% in Italy and Canada [16,19,34]. However, no studies have investigated the occurrence of CanineCV in golden jackals despite their wide distribution across Eurasia and ongoing expansion [2]. The only available study involving a similar species was performed in Namibia by de Villiers et al. [14], documenting an infection frequency in black-backed jackals that was slightly lower, approximately 40%, compared to our results. Research so far suggests that CanineCV genotype 5 has been identified exclusively in wildlife samples, while other CanineCV genotypes can be detected in both domestic dogs and wild canids [14,16,19]. Interestingly, our study shows an infrequent finding suggesting that foxes may also harbor other genotypes beyond genotype 5 [13]. Although genotype 5 is generally more prevalent in fox populations, our findings challenge the claim made by Magliocca et al. [35] regarding its limited transmissibility between different animal species. Instead, our results suggest the circulation of both CanineCV genotypes among the examined wild carnivore populations. This is further supported by the clustering of these strains in the phylogenetic tree as well as by the environmental resistance of circoviruses conducive to indirect transmission between these populations [12].

Prior examinations show that CanineCV strains do not group phylogenetically based on animal species but display geographical clustering to a certain extent, with Asian sequences mainly forming separate genotypes, as verified by our results and the results of other researchers [17,18,36]. The sequences from this study that clustered within genotype 5 were distinct, indicating individual transmission cycles within different locations. Nevertheless, one jackal-derived strain (PP493390) was separated from other Serbian strains aligning with the results obtained by recombination and haplotype diversity analyses. Canuti et al. [19] demonstrated the separation of CanineCV genotype 5 representatives into four clades, mostly based on geographic distribution. Our results add to these data by displaying a separate clade with Serbian sequences from jackals and one fox. Interestingly, a sequence from neighboring Croatia clustered with Canadian representatives in clade 5, rather than with our sequences [15]. Furthermore, as described by Franzo et al. [16], Italian genotype 5 strains showed a closer relationship to those from Norway and the UK than to Croatian and even other Italian viruses. This further suggests different transmission pathways in wildlife across the Balkans, despite our sampling locations being approximately 240 km apart by air distance. Even though the directions of viral transmission in wild carnivores should be viewed in relation to host movements, geographical barriers between Serbia and Croatia, such as rivers, may contribute to the separation of wild carnivore populations. Nevertheless, further research is needed to confirm this, as there is no recent data on CanineCV in wildlife across the Balkans.

Nucleotide similarities above 80% noted among all sequences from this study, as well as with other GenBank representatives, were agreeable with the recently updated species demarcation threshold for circoviruses [20]. However, this value was previously questioned by Franzo et al. [16] due to the clustering of CanineCV genotype 5 sequences and their high genetic diversity. Having in mind that the *Cap* protein is a key structural component involved in stimulating the immunological response in the host, we focused on its analysis in the detected CanineCV strains [12,21]. Our results concerning the nucleotide and amino acid variability of the *Cap* gene and protein sequences are comparable to those reported by Sun et al. [37] and Dankaona et al. [21], exhibiting variability in this region. However, our results suggest greater genetic diversity within genotype 5 compared to genotype 4, possibly due to different evolutionary pressures such as ongoing adaptation to jackal hosts or host immune pressures [11]. It should also be noted that we found mixed infections with both genotypes in jackal-derived samples, indicating that these animals may act as reservoirs or bridging hosts enabling potential recombination between the two genotypes contributing to genetic variability and adaptation. Although we observed numerous amino acid variations, strong purifying selection suggests that most non-synonymous mutations are eliminated. This trait has been described in other studies and provides the structural and functional integrity of the *Cap* protein [21,37]. Also, the observed higher rate of transitions over transversions, typical of conservative evolution, reduces the likelihood of disruptive mutations. Nevertheless, one site was identified under positive selection, potentially denoting a region under immune pressure or subject to host-specific adaptation [21,36].

The detected recombination events in CanineCV strains provide some insights into their evolutionary mechanisms, especially in the context of the detected mixed infections in jackals. The potential recombination breakpoint between the *Rep* and *Cap* genes implies that genetic exchange between viral strains may impact replication efficiency and capsid-mediated host immune interactions. Our results are in agreement with some previous studies showing that recombination events in circoviruses are not restricted to the *Rep* gene and can occur in other regions as well [9,31,37,38]. Having in mind that purifying selection is dominant in the *Cap* gene, recombination might be an alternative means for introducing genetic diversity while preserving overall functional constraints. Interestingly, de Villiers et al. [14] also noted CanineCV sequences from black-backed jackals in Namibia as recombinant, with strains circulating in domestic dogs serving as parental strains. Although recombination events were detected and mixed infections occurred in jackals, the absence of CanineCV data from sympatric domestic dog populations (both in this study and in available regional literature) limits our ability to infer transmission direction or potential domestic origins of recombinant strains. Given the increasing overlap of wild carnivores and domestic dogs in peri-urban areas of Serbia, this would be compelling to examine. Jackals may serve as recombination hotspots allowing for the emergence of novel viral variants considering that one jackal-derived strain had a specific recombination pattern. However, this single finding does not allow for definitive conclusions and is a key limitation of our study that warrants further investigation. Genetic exchange facilitating viral adaptation is enabled through interspecies transmission in wild carnivore populations, along with the occurrence of mixed infections [36,37]. The presence of mixed infections with different circovirus strains, previously noted for PCV2, results in inter- and intra-genotype recombination and is also connected to increased disease severity [39]. Considerable haplotype diversity of CanineCV strains in this study further supports the results of phylogenetic and recombination analyses. High genetic variation was determined among the analyzed sequences, and no dominant haplotype emerged [21]. Furthermore, jackal-derived CanineCV sequences grouped with Italian dog and wolf-derived sequences, implying possible historical connection across regions. CanineCV genotype 5 sequence clustering indicated these are geographically widespread but related lineages, as shown by phylogeny [32]. The recombinant jackal-derived sequence emerged as the most divergent haplotype (Hap_131, PP493390), while the fox sequence (Hap_137, PP493396) formed a separate branch, supporting the concept of host-specific variants [19].

## 5. Conclusions

The presented results add to the growing number of studies concerning the global distribution and diversity of CanineCV. Aside from the noted high infection frequency in wild carnivores, especially golden jackals, these animals were highlighted as hosts for mixed infections with distinct viral genotypes. Phylogeny and haplotype analysis supported the evidence that these viruses are genetically diverse and mostly cluster based on a geographical pattern and under the influence of viral transmission within populations. However, due to high sequence variation, there was no dominant haplotype, and a jackal-derived sequence with a distinct recombination pattern stemmed as a divergent lineage indicative of a novel evolutionary pathway. Viral integrity is maintained through the observed dominance of purifying selection in the *Cap* gene, with only one site detected to be under immune pressure. However, genetic recombination is a possible mechanism to introduce genetic diversity in CanineCV strains and the detected multiple recombination events between the *Rep* and *Cap* genes show these changes may have functional implications and contribute to enhanced viral adaptability. The appearance of mixed circovirus infections in jackals, as well as positive PCR signals for both general CanineCV and genotype 5 in samples from foxes, point to potential interspecies viral transmission. This is further supported by the identification of recombinant strains through sequence analysis. Additionally, jackals were marked as potential recombination hotspots for CanineCV, that may enable the emergence of novel viral variants. However, no data from domestic dogs were included in this study, limiting our ability to evaluate potential domestic–wild transmission dynamics or identify the origin of recombinant strains. The presented results deliver new data concerning mechanisms of CanineCV evolution, indicating the presence of complex selection pressures and host-driven differentiation under geographical dispersal patterns. Ongoing surveillance is essential to track the evolutionary pathways of CanineCV and its potential to adapt to various hosts. Also, the investigation should be expanded to include parallel sampling of both wild and domestic carnivores, especially those from peri-urban areas, where there is frequent interaction.

## Figures and Tables

**Table 1:** Real-time PCR results relative to wild carnivore species sampled and the proportion of single and mixed infections.

	CanineCV	CanineCV Genotype 5	Mixed Infections
Jackals	9/19 (47.4%)	6/19 (31.6%)	4/19 (21%)
Foxes	2/12 (16.7%)	10/12 (83.3%)	/
Ʃ	11/31 (35.5%)	16/31 (51.6%)	4/31 (12.9%)

**Table 2:** Substitution rates under the GTR + G + I model for the *Cap* gene of analyzed sequences. Each entry is the probability of substitution (*r*) from one base (row) to another base (column). Rates of different transitional substitutions are shown in bold and those of transversionsal substitutions are shown in italics.

From\To	A	T	C	G
A	-	*7.1847*	*5.3200*	**14.4596**
T	*10.6413*	-	**12.6742**	*2.3790*
C	*6.6378*	**10.6769**	-	*3.0859*
G	**21.0123**	*2.3341*	*3.5941*	-

**Table 3:** Selection pressure analysis of *Cap* protein of CanineCV strains using SLAC, MEME, FEL, and FUBAR methods. The bold formatting denotes statistically significant results.

Codon	SLAC		MEME		FEL		FUBAR	
	dN − dS	*p*-Value	β+	*p*-Value	dN − dS	*p*-Value	dN − dS	Post. pr.
29	4.25	0.34	0.26	0.59	1.62	0.17	3.435	**0.90**
83	5.37	0.17	15.71	**0.06**	2.30	**0.05**	6.031	**0.97**

## Data Availability

All data generated or analyzed during this study are included in this published article [and its Supplementary Information Files].

## Supplementary Materials

The following supporting information can be downloaded at: https://www.mdpi.com/article/10.3390/vetsci12060515/s1, Table S1: Amino acid variations in the *Cap* protein of CanineCV genotype 4 strains from wild carnivores; Table S2a,b: Amino acid variations in the *Cap* protein of CanineCV genotype 5 strains from wild carnivores.
