# Supplementary material for: Genetic Diversity of Canine Circovirus Detected in Wild Carnivores in Serbia
Source: Vet Sci. 2025 May 24;12(6):515. doi: 10.3390/vetsci12060515 (PMC12197379; doi:10.3390/vetsci12060515)
Supplement: Supplementary file 1 [file vetsci-12-00515-s001.zip › vetsci-3616397-supplementary.pdf]

Supplementary Table S1 Amino acid variations in the *Cap* protein of CanineCV genotype 4 strains from wild carnivores

	**13**	**16**	**17**	**32**	**44**	**50**	**79**	**83**	**85**	**86**	**89**	**94**	**100**	**102**	**105**	**111**	**116**	**145**	**148**	**195**	**198**	**240**	**242**	**243**
**NC_020904***	S	T	R	L	D	V	A	T	D	F	L	F	V	I	R	R	N	V	R	A	S	D	S	T
**PP493391**						M		V	N	Y	P	T	I		K	K	D		S	T		E	T	
**PP493392**	R		H		G		T									T			I	T				
**PP493393**		S		M									I	V	K			T		T	T			N
**PP493395**							T								K	K		T		T	T			N

*Reference strain

**The amino acids corresponding to the single-letter codes: S (Serine), T (Threonine), R (Arginine), L (Leucine), D (Aspartic acid), V (Valine), A (Alanine), F (Phenylalanine), I (Isoleucine), N (Asparagine), M (Methionine), Y (Tyrosine), P (Proline), K (Lysine), E (Glutamic acid), H (Histidine), and G (Glycine).

Supplementary Table S2a Amino acid variations in the *Cap* protein of CanineCV genotype 5 from wild carnivores

	**13**	**14**	**16**	**19**	**20**	**28**	**29**	**32**	**44**	**50**	**79**	**83**	**85**	**86**	**89**	**94**	**95**	**100**	**102**	**103**	**105**	**113**	**116**	**128**	**138**	**144**	**145**
**KP260927***	R	N	P	Y	S	R	N	L	D	I	A	V	N	Y	P	Y	F	V	V	K	K	M	N	E	S	A	V
**PP493390**		Y	T	L	N	N	R	M	G			I	D	F		S	Y	I	I	R	R	L	D			T	
**PP493394**		Y	S			N				M						F			I	R	R	L		V	Y		
**PP493396**		Y	S			N				V									I	R		L					
**PP493397**	S	Y	T	L	N	N				V		T	D	F		F	Y		I	R		L				T	T
**PP493398**	S	Y	T	L	N	N	R	M		M	T	I	D	F	P	S	Y		I	R	R	L				T	T

*Reference strain

**The amino acids corresponding to the single-letter codes: R (Arginine), N (Asparagine), P (Proline), Y (Tyrosine), S (Serine), L (Leucine), D (Aspartic Acid), I (Isoleucine), A (Alanine), V (Valine), K (Lysine), M (Methionine), E (Glutamic acid), T (Threonine), G (Glycine), F (Phenylalanine).

Supplementary Table S2b Amino acid variations in the *Cap* protein of CanineCV genotype 5 from wild carnivores

	**148**	**149**	**159**	**169**	**193**	**194**	**195**	**201**	**208**	**214**	**228**	**231**	**234**	**236**	**239**	**240**	**242**	**245**	**257**	**267**
**KP260927***	T	T	P	A	D	V	S	A	Q	M	L	M	M	S	P	E	S	P	V	G
**PP493390**	R	L		S		I	T		T	I				P		D			I	D
**PP493394**	R	M	T	S	E		A	T		R	I		V	H	Q		F	Q	I	
**PP493396**	R	M			E		A		T	I			V	H	Q		F	Q	I	
**PP493397**	R	L		S	E		A		T	I		I		P				Q	I	
**PP493398**	R	L		S	E		A		T	I			V	H	Q		F	Q	I	

*Reference strain

**The amino acids corresponding to the single-letter codes: T (Threonine), P (Proline), A (Alanine), D (Aspartic acid/Aspartate), V (Valine), S (Serine), Q (Glutamine), M (Methionine), L (Leucine), E (Glutamic acid/Glutamate), G (Glycine), R (Arginine), I (Isoleucine), H (Histidine), and F (Phenylalanine).
